# MicroRNAs in Different Histologies of Soft Tissue Sarcoma: A Comprehensive Review

**DOI:** 10.3390/ijms18091960

**Published:** 2017-09-12

**Authors:** Maria Anna Smolle, Andreas Leithner, Florian Posch, Joanna Szkandera, Bernadette Liegl-Atzwanger, Martin Pichler

**Affiliations:** 1 Department of Orthopaedics and Trauma, Medical University of Graz, 8036 Graz, Austria; maria.smolle@cbmed.at (M.A.S.); andreas.leithner@medunigraz.at (A.L.); 2 Division of Oncology, Department of Internal Medicine, Medical University of Graz, 8036 Graz, Austria; florian.posch@medunigraz.at (F.P.); joanna.szkandera@medunigraz.at (J.S.); 3 Institute of Pathology, Medical University of Graz, 8036 Graz, Austria; bernadette.liegl-atzwnager@medunigraz.at

**Keywords:** soft tissue sarcoma, microRNAs, tumourigenesis

## Abstract

Soft tissue sarcomas (STS) constitute a rare tumour entity comprising over 50 histological subtypes. MicroRNAs (miRNAs) are short non-protein coding RNA molecules that regulate gene expression by targeting the 3’-untranslated region of messenger RNAs. They are involved in a variety of human diseases, including malignancies, such as endometrial cancer, osteosarcoma, bronchial carcinoma and breast cancer. In STS, various miRNAs are differentially expressed, thus contributing to development, progression and invasion. Therefore, the aim of the present review is to summarise current knowledge on the role of miRNAs in STS. Furthermore, the potential role of miRNAs as diagnostic, prognostic and predictive biomarkers is discussed.

## 1. Introduction

Soft tissue sarcoma (STS) is a rare tumour entity, with an estimated incidence of 4–5 per 100,000 per year [1]. STS may occur anywhere in the human body, from the extremities and trunk to the viscera and the retroperitoneum. The diagnosis is aggravated by atypical symptoms of variable duration. Consequently, unplanned excisions of STS, deemed benign, are not uncommon and may occur in up to 40% of patients [2]. Over 50 different subtypes of STS can be distinguished, with the most common types being liposarcoma, leiomyosarcoma and undifferentiated pleomorphic sarcoma [3]. Treatment of STS is complex and consists of wide en-bloc resection, combined with chemotherapy (CTX) and radiotherapy (RTX) in select cases [4]. In contrast to other malignancies, and especially those of epithelial origin, many STS subtypes show poor responses to CTX, which could be related to the fact that different biological processes drive tumour growth and metastasis in mesenchymal neoplasms [5]. Different clinico-pathological factors as well as laboratory biomarkers have been proposed as potential prognostic factors, but due to the heterogeneity of underlying biology, many of them lack generalizability and reliability [6].

MicroRNAs (miRNAs) are small non-protein coding RNA molecules, with a length of 21–25 nucleotides, that exert regulatory functions on gene expression. By binding—in the context of the RNA-induced silencing complex (RISC)—to the 3′-untranslated region (3′UTR) of target mRNA, miRNAs cause degradation of the respective mRNA and/or translation inhibition [7]. Their genes are both located in intergenic and intronic regions [8]. Via complex nuclear and cytoplasmic mechanisms, primary miRNAs (pri-miRNAs) are modified into precursor miRNAs (pre-miRNAs) [9]. Finally, the cytoplasmic RNAse-III enzyme, Dicer, produces mature miRNAs out of the hairpin-shaped pre-miRNAs [10].

Dysregulation of specific miRNAs has been reported in many human malignancies [11,12], including, for example, endometrial cancer [13], acute myeloid leukaemia [14], breast cancer [15,16], diffuse large B-cell lymphoma [17], colorectal cancer [18,19,20], bronchial carcinoma [21], testicular cancer [22], and osteosarcoma [23]. miRNAs can be involved in the pathogenesis, maintenance or progression of malignancies. They can serve as tumour suppressors—and are consequently downregulated in the respective malignancy—or exert oncogenic functions as overexpressed miRNAs [11]. The dysfunction of miRNAs in human cancer is caused by similar events to proteins: a deficiency in the processing pathway [24], epigenetic modifications [25] or miRNA gene mutations [26] all alter miRNA expression.

In clinical oncology, miRNAs can have diagnostic, prognostic and predictive significance and may even be used as therapeutic targets [27].

**Figure 1:**
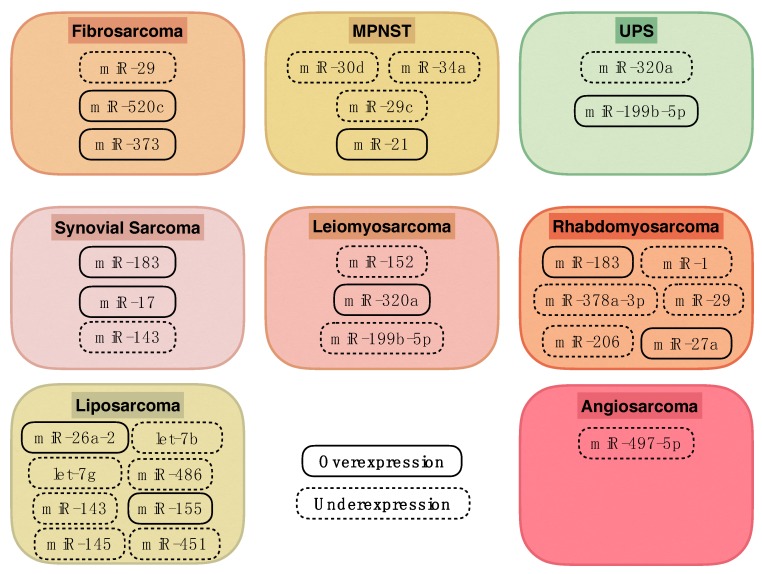
miRNA expression in different soft tissue sarcoma (STS) subtypes.

In the present review, the role of miRNAs in different STS subtypes will be highlighted (Figure 1, Table 1). Moreover, their evolving role in clinical practice will be discussed. As different histologic subtypes of STS considerably differ in their clinical behaviours and underlying pathobiologies, the management of STS is evolving towards a histotype-specific approach. Our review follows this model by discussing miRNAs within histologic STS subentities. The choice of literature discussed was based on original works describing correlations between miRNAs and distinct STS subtypes.

## 2. MicroRNAs in Human Soft Tissue Sarcoma

### 2.1. Liposarcoma

Liposarcomas account for approximately 15% of all STS [3]. Depending on their histological appearance, liposarcomas can be subdivided into pleomorphic, myxoid/round cell, dedifferentiated and well-differentiated liposarcomas [28].

In almost 90% of well- and dedifferentiated liposarcomas, an amplification of the *12q13-q22* gene region is present, as well as an overexpression of mouse double minute 2 homolog (MDM2) and cyclin dependent kinase 4 (CDK4), proteins characteristic of these liposarcoma subtypes [29]. Additionally, miR-26a-2, which is located near the *MDM2* gene region is overexpressed in well- and dedifferentiated liposarcomas [30]. This overexpression results in enhanced cellular proliferation, survival and invasion [30]. On the other hand, the experimental overexpression of the miR-26a-2 target regulator of chromosome condensation, and BTB domain containing protein 1 (RCBTB1), results in enhanced susceptibility to apoptosis [30]. Homeobox protein A5 (HOXA5), which is downregulated in liposarcoma, constitutes another target of miR-26a-2 [31]. By inhibiting miR-26a-2 expression and concurrent HOXA5-overexpression, the susceptibility of liposarcoma cells to p-53 independent apoptotic stimuli, significantly increases [31]. Consequently, the repression of miR-26a-2 may be used in clinical practice to reduce liposarcoma growth and progression.

miR-155 is significantly overexpressed in myxoid/round cell-, dedifferentiated-, and pleomorphic-liposarcomas, as compared with normal adipose tissue [32]. It is a strong oncogene and promotes cellular growth by targeting casein kinase 1α (CK1α), which in turn enhances β-catenin signalling and the expression of cyclin D1 [32]. The knockdown of miR-155 results in reduced tumour growth, in vitro and in vivo, as the Gap1 (G1)-synthesis (S) cell-cycle progression is blocked [32]. In human plasma samples, miR-155 levels allow a distinction between healthy individuals, patients with benign lipomas and those suffering from dedifferentiated liposarcomas [33].

In myxoid liposarcoma, a subtype characterised by the t(12;16)(q13;p11) translocation, miR-486 is significantly downregulated [34]. This miRNA interacts with the plasminogen activator, inhibitor-1 (PAI-1), an important promoter of cellular proliferation and invasion [35]. Exogenous overexpression of miR-486 is associated with the reduced growth of tumour cells in myxoid liposarcoma [34]. Consequently, miR-486 may represent a potential therapeutic avenue for reducing cellular proliferation in this sarcoma entity.

In well-differentiated and dedifferentiated liposarcomas, miR-143 is significantly downregulated, in comparison to normal adipose tissue [36]. As the expression levels of miR-143 decrease, from well- to dedifferentiated liposarcomas, this miRNA seems to be already involved in the early processes of tumourigenesis [36]. Apoptosis is induced, and cellular proliferation diminished, upon experimental overexpression of miR-143 in dedifferentiated liposarcoma. Additionally, expression levels of polo-like kinase 1 (PLK1), protein regulator of cytokinesis 1 (PRC1), B-cell lymphoma 2 (BCL2) and topoisomerase 2A, decrease [36]. Both PRC1 and PLK1 are involved in cytokinesis [37], explaining the inhibitory effect of miR-143 expression on cellular proliferation.

Moreover, the miR-143/miR-145 cluster (gene region *5q32*) is downregulated along with the miR-144/451 (gene region *17q11.2*) cluster in liposarcoma, in contrast to benign adipocytic tumours [38]. The experimental overexpression of miR-145 and miR-451 results in diminished cellular proliferation, impairs cell cycle progression and induces apoptotic cell death [38].

In well- and dedifferentiated liposarcomas, two members of the let-7 family of miRNAs—let-7b and let-7g—are inhibited, in contrast to benign adipocytic tumours [39]. Moreover, the inhibition of let-7 miRNA may be involved in the deregulation of *high-mobility group AT-hook2 (HMGA2)*, whose rearrangement is typically found in benign, as well as malignant, adipocytic tumours, since the 3′UTR of *HMGA2* contains multiple-binding sites for the let-7 family [40,41]. According to recent investigations, however, the inhibition of let-7 family members does not seem to be the main event responsible for *HMGA2* dysregulation, nor HMGA2 protein overexpression [39].

### 2.2. Leiomyosarcoma

Leiomyosarcomas are defined as demonstrating a smooth muscle phenotype with immunohistochemistry and account for approximately 10% of all STS [42]. Leiomyomas, their benign counterparts, occur far more frequently.

In leiomyosarcoma, miR-152 is downregulated [43]. This miRNA has been found to be involved in various malignancies, including bladder and prostate cancer [44,45]. miR-152 has several targets, such as DNA (cytosine-5)-methyltransferase-1 (DNMT1) in endometrial cancer and TNFRF6B in hepatocellular carcinoma. In leiomyosarcoma, miR-152 targets the tyrosine-protein kinases: MET and KIT [43]. MET plays a role in cellular invasion by binding hepatocyte growth factor (HGF) and by activating signalling pathways involved in cellular migration [46]. KIT acts as a promoter in tumourigenesis via the activation of downstream pathways that result in cellular proliferation [47]. The downregulation of miR-152 in leiomyosarcoma is associated with increased KIT and MET-levels [43]. On the other hand, experimental upregulation of miR-152 reduces levels of MET and KIT in leiomyosarcoma cell lines [43]. Moreover, miR-152 diminishes proliferation and enhances apoptotic cell death, reduces the activity of the PI3K/AKT cascade and leads to S phase cell cycle arrest [43].

As mentioned above, the molecular differentiation between UPS and leiomyosarcoma can be difficult. In this respect, miRNA signatures have emerging potential as diagnostic biomarkers for aiding subclassification. For example, miR-199b-5p-levels are significantly higher in UPS as compared with leiomyosarcoma, while miR-320a is upregulated in leiomyosarcoma, whilst being downregulated in UPS [48]. Consequently, these miRNAs may not only serve as diagnostic markers, but also as therapeutic targets to influence tumour development and progression.

### 2.3. Synovial Sarcoma

In contrast to most STS that mainly occur in adults, synovial sarcomas generally arise in children, teenagers and young adults [49]. They are characterised by fusion of the *Synovial Sarcoma Translocation* (*SYT*) gene, located on chromosome 18, with Synovial Sarcoma, X breakpoint 1, 2 or 4 (SSX1, SSX2, SSX4) on the X chromosome [50].

miR-183, located on chromosome 7, is overexpressed in synovial sarcoma as well as in rhabdomyosarcoma and colon cancer [51,52]. It targets the tumour suppressors—early growth response protein 1 (EGR1) and phosphatase and tensin homolog (PTEN)—and blocks their translation [51]. Moreover, EGR1 is repressed by the SS18-SSX fusion gene in synovial sarcoma [53]. The knockdown of miR-183 results in the enhanced expression of EGR1 and PTEN mRNAs as well as protein levels. Additionally, the knockdown of miR-183 is associated with decreased tumour cell migration [51]. In clinical practice, the targeting of miR-183 may result in enhanced EGR1 and PTEN-levels, thus reducing cellular migration and invasion.

Another miRNA that is overexpressed in synovial sarcoma is miR-17, which is induced by the *SS18-SSX* fusion gene and is organised in a cluster (miR-17-92 cluster) [54,55]. It acts as an oncogene by directly impairing the expression of cyclin-dependent kinase inhibitor 1 (p21, CDKN1A), a cyclin-dependent kinase inhibitor, which regulates the transition from the G1 into the S-phase of the cell cycle [56]. Colony formation and cellular growth are enhanced by miR-17 overexpression, whilst cellular mobility and invasion remain unaffected [54]. On the other hand, experimental blockage of miR-17 leads to increased p21-levels, and thus, reduced cell proliferation [54].

Recently, a panel of seven blood-borne miRNAs, measured in the peripheral blood, was able to discriminate synovial sarcoma patients from healthy individuals and patients with leiomyosarcoma, Ewing sarcoma, malignant peripheral nerve sheath tumour (MPNST) and liposarcoma [57]. The seven miRNAs which are upregulated in synovial sarcomas include miR-99a-5p, -146b-5p, -148b-3p, -195-5p, -223-3p, -500b-3p and -505-3p [57]. By testing synovial sarcoma patients for this panel of upregulated miRNAs, even earlier detection of local recurrence and distant metastasis could be made possible [57].

As with well- and dedifferentiated liposarcomas, miR-143 is significantly downregulated in synovial sarcoma [58]. SSX1 is a potential target for miR-143, as predicted by *miRBase* and *Target Scan 3.1.* As the fusion protein SYT-SSX1 is characteristic of synovial sarcoma, miR-143 downregulation may contribute to formation of this oncoprotein [58].

### 2.4. Malignant Peripheral Nerve Sheath Tumour (MPNST)

Malignant peripheral nerve sheath tumours (MPNSTs) constitute a rare subtype of STS. They usually arise from large peripheral nerves and are associated with Neurofibromatosis Type 1 (NF1) in up to 50% of patients [59]. Due to their often complicated location, necessitating wide resection, and their high metastatic potential, the prognosis of MPNST is rather poor [60].

In MPNST, the histone methyltransferase, enhancer of zeste homolg 2 (EZH2), is significantly upregulated, in comparison to healthy nerve tissue and neurofibromas [61]. EZH2 forms the polycomb-repressor complex 2 (PRC2) with two other molecules and thus represses gene transcription [62]. However, the PRC2 complex itself is frequently inactivated in MPNST, due to mutations in the *SUZ12* and *EED* gene regions, which encode parts of the complex [63,64]. Therefore, other mechanisms than the formation of the PRC2 complex may promote MPNST-tumourigenesis. For example, EZH2 directly targets the promoter region of miR-30d, and thus represses its transcription [61]. miR-30d itself usually targets importin subunit beta-1 (KPNB1, encoded by KPNB1) and suppresses its translation. The knockdown of *KPNB1* results in enhanced apoptosis of MPNST cells, an effect also observed with *EZH2*-knockdown and miR-30d overexpression [61]. Importantly, the overexpression of EZH2 and KPNB1 in MPNST is negatively correlated with miR-30d expression [61]. Therefore, the EZH2–miR-30d–KPNB1-axis could serve as a target in anticancer therapy for patients with MPNST.

Another potential therapeutic target is the p53–miR-34a interaction. miR-34a usually regulates many genes involved in proliferation and cell cycle progression [65]. Moreover, it is upregulated by the tumour suppressor, p53 [66]. In the majority of MPNSTs, p53 is inactivated, in contrast to benign neurofibroma [67]. Consequently, miR-34a is also downregulated [67]. By adding wt-p53-GFP-containing plasmids to MPNST cell lines, miR-34a levels significantly increase [67]. Moreover, both the overexpression of p53 and miR-34a promote apoptotic cell death of MPNST cells in vitro [67]. Taking into account that a phase 1 clinical trial has already tested the restitution of miR-34 via MRX34 as an anticancer therapeutic, miR-34a substitution may also be of value in MPNST [68,69]. Notably though, the trial had to be terminated due to immune-related adverse events, indicating that some adaptations still need to be performed.

miR-29c is another miRNA which is downregulated in MPNST, in contrast to benign neurofibroma [70]. It targets extracellular matrix genes and matrix metalloproteinase (MMP)-2, which play significant roles in cell migration and tumour invasion, by degrading collagens and glycoproteins in the extracellular matrix [71]. The experimental upregulation of miR-29c results in reduced cellular invasion, whilst cell proliferation remains unchanged [70]. Concurrently, higher miR-29c levels correlate with lower activity of MMP-2 in MPNST cell lines [70]. Therefore, therapeutic administration of miR-29c may be a worthwhile strategy to impair the invasive and metastatic potential of MPNST.

Conversely, miR-21 is upregulated in MPNST, in contrast to neurofibroma [72]. Its target, programmed cell death protein 4 (PDCD4), normally acts as a tumour suppressor by inducing apoptosis via a caspase cascade [73]. Neurofibromas and normal peripheral nerves express PDCD4 at significantly higher levels than MPNSTs [72]. At the same time, the knockdown of miR-21 in MPNST cell lines results in enhanced expression of PDCD4 [72]. Consequently, the blockage of miR-21 could be used in clinical practice to induce tumour cell apoptosis.

### 2.5. Rhabdomyosaroma

Rhabdomyosarcoma constitutes the most frequent STS type in the paediatric population and can be subdivided into alveolar, embryonal, pleomorphic and spindle cell/sclerosing rhabdomyosarcoma. Whilst the molecular features of the latter are not well understood, alveolar rhabdomyosarcomas are characterised by the fusion between *PAX7* or *PAX3*, and *FOXO1A* [74,75].

As with synovial sarcoma, miR-183 is also overexpressed in rhabdomyosarcoma [51]. The knockdown of this miRNA leads to increased mRNA and protein levels of EGR1 and PTEN, two tumour suppressors [51]. Moreover, cellular migration is mitigated by the knockdown of miR-183; therefore, it could serve as a therapeutic target in the treatment of rhabdomyosarcoma.

miR-378a-3p, a member of the miR-378 family, is significantly downregulated in rhabdomyosarcoma, as compared with healthy skeletal muscle [76]. It targets insulin-like growth factor receptor 1 (IGF1R), an important component of the IGF1R/AKT-signalling pathway. This pathway is known to be involved both in cellular proliferation and myogenic differentiation [77]. Experimental overexpression of miR-378a-3p does not only lead to reduced expression of IGF1R but also results in increased apoptosis via the blockage of phospho-AKT and induction of caspase-3 [76]. Additionally, the presence of miR-378a-3p is associated with cell cycle arrest in the G2 phase [76]. Therefore, this miRNA might serve as a therapeutic target in rhabdomyosarcoma, with the administration of miR-378a-3p-mimics reducing cellular proliferation and enhancing apoptotic cell death.

miR-1 and mir-206 are further miRNAs that are downregulated in both embryonal and alveolar rhabdomyosarcoma [78]. They repress the protein expression of PAX3 in embryonal, but not alveolar, rhabdomyosarcoma [78]. This discrepancy may be explained by the fact that the *PAX3-FOXO1A* fusion gene is present in alveolar rhabdomyosarcoma, whilst the embryonal subtype is lacking this gene fusion. Therefore, the *PAX3-FOXO1A* fusion might hamper the regulation of PAX3 by miRNAs in alveolar rhabdomyosarcoma.

Moreover, miR-1, -206 and -29 usually target G1/S-specific cyclin-D2 (CCND2), a cell-cycle protein found to be upregulated in several malignancies, and repress its translation [78,79]. Additionally, miR-29 also targets transcription factor E2F7, which is involved in DNA repair and replication, mitosis and cell cycle regulation [78,80]. Consequently, miR-29, miR-1 and miR-206 have potential tumour-suppressive functions; therefore, their mimics may be used in the treatment of rhabdomyosarcoma.

A miRNA that is upregulated in rhabdomyosarcoma is miR-27a. It is specifically found in the aggressive, translocation-positive alveolar subtype [81]. There, it promotes cellular proliferation and reduces the number of cells in the cell cycle’s G-phase. Moreover, miR-27a targets retinoic X receptor alpha (RXRA) and retinoic acid receptor alpha (RARA). Retinoic acid receptors (RARs) usually interact with retinoic X receptors (RXRs) as heterodimers, and act as growth inhibitors by regulating transcription via bondage to retinoic acid response elements (RAREs) [82]. Upon experimental overexpression of miR-27a, levels of RXRA and RARA significantly decrease [81]. Therefore, miR-27a has oncogenic functions in rhabdomyosarcoma and its blockage could be used in practice to lessen tumour cell growth.

### 2.6. Fibrosarcoma

Fibrosarcoma constitutes a rare subtype of STS. Its incidence has constantly decreased over the years, due to the improved pathologic discrimination of fibrosarcoma subtypes and distinction from fibrosarcoma-like mesenchymal neoplasms [83].

The miR-29 family is not only involved in the pathogenesis of MPNST, but also contributes to the progression and invasion of fibrosarcoma cells [84]. Reduced invasion of fibrosarcoma cells can be achieved by experimental overexpression of miR-29 family-members [84]. The impairment of invasion is facilitated by a downregulation of *MMP2*, a matrix-metalloproteinase that is important for modulation of the extracellular matrix [85]. In clinical practice, the administration of miR-29 could therefore lessen the invasive and metastatic potential of fibrosarcomas.

miR-520c and miR-373 are two miRNAs which are also involved in the modulation of the extracellular matrix of fibrosarcomas [86]. They indirectly upregulate the expression of *matrix-metalloproteinase (MMP)-9* by targeting the 3′-UTR of mechanistic target of rapamycin (mTOR) and sirtuin 1 (SIRT1) [86]. As a result, the Ras–Raf–MEK–Erk signalling pathway is activated, and phosphorylates the nuclear factor kappa-light-chain-enhancer of activated B cells (NF-κb), leading to enhanced transcription and translation of MMP9 [86]. Consequently, cellular growth and migration is enhanced [86].

### 2.7. Undifferentiated Pleomorphic Sarcoma (UPS)

UPS—formerly termed malignant fibrous histiocytoma (MFH)—is the most common type of STS in adults over the age of 40. However, UPS is an exclusion diagnosis, only made after thorough analysis of the histological specimen using modern technologies [75].

As with leiomyosarcoma, miR-152 is downregulated in UPS and negatively correlates with MET and KIT mRNA levels [43]. Moreover, experimental upregulation of miR-152 results in reduced KIT and MET mRNA as well as protein levels, down-regulates the PI3K/AKT-pathway, diminishes cellular growth and enhances apoptotic cell death [43].

Although leiomyosarcoma is characterised by smooth muscle differentiation and is usually positive for desmin, h-caldesmon and smooth muscle actin (SMA), it lacks recurrent genetic alterations [87]. On the other hand, UPS is defined as an STS lacking any precise cellular differentiation. However, in UPS, recurrent genetic features are also missing [88]. Contrary to leiomyosarcoma, miR-199b-5p is significantly upregulated in UPS [48]. Additionally, levels of miR-320a are significantly lower in UPS compared with leiomyosarcoma [48]. Therefore, a distinction between leiomyosarcoma and UPS is possible, through the analysis of miRNA-patterns.

### 2.8. Angiosarcoma

Angiosarcoma is a highly aggressive subtype of STS, with about half of patients dying from the disease within one year following diagnosis [75]. The development of angiosarcoma is sometimes associated with lymphoedema (e.g., following radical axillary lymph node resection). Most commonly, angiosarcomas present as cutaneous neoplasms rather than deep-seated STS [75].

miRNAs that are downregulated in angiosarcoma are miR-497-5p, -378-3p and 483-5p, of which miR-497-5p interacts with the potassium intermediate conductance calcium-activated channel KCa3.1 [89,90]. This channel is involved in cancer cell growth and invasion, and is upregulated in angiosarcoma [90]. Experimental upregulation of miR-497-5p results in reduced levels of KCa3.1 and impairs cellular proliferation, progression of the cell cycle and cellular invasion. This effect is achieved by the downregulation of cyclin D1, survivin and p53, which are all involved in the regulation of the cell cycle [90]. In clinical practice, miR-497-5p could be used as a therapeutic agent, directly targeting KCa3.1, and thus preventing tumour progression and invasion.

Other than miR-497-5p, the miR-17-92 cluster is significantly upregulated in MYC proto-oncogene (MYC)-amplified angiosarcoma [91]. Of note, whilst secondary angiosarcomas that arise due to prior irradiation usually carry an amplification of the oncogene *MYC*, this alteration is less common in primary angiosarcomas [92]. MYC is involved in cellular differentiation, growth and apoptosis, and plays a role in various human cancers [93]. The upregulation of the miR-17-92 cluster is associated with the downregulation of thrombospondin 1 (THBS1), an adhesive glycoprotein, which mediates cell–matrix and cell–cell interactions, with the potential to inhibit angiogenesis [94]. Therefore, the miR-17-92 cluster could be therapeutically targeted to lessen angiogenetic tumour growth in MYC-positive angiosarcomas.

## 3. Conclusions

Distinct miRNAs are differentially expressed in various soft tissue sarcoma subtypes. Depending on the miRNA and tumour type, they have diagnostic, prognostic, predictive and therapeutic potential. Yet, the usage of miR-inhibitors or mimics in clinical practice is still under investigation and has not reached clinical routine. Nevertheless, the targeting of miRNAs or administering of miRNA-mimics may pose promising therapeutic strategies in soft tissue sarcomas that show poor responses to conventional chemotherapy.

## Figures and Tables

**Table 1:** Expression pattern of miRNAs in different STS-subtypes and their target mRNAs.

microRNA	Histological Subtype	Expression	Target
*let-7b*	Well-/dedifferentiated liposarcoma	Underexpression	
*let-7g*	Well-/dedifferentiated liposarcoma	Underexpression	
*miR-1*	Rhabdomyosarcoma	Underexpression	CCND2, PAX3
*miR-143*	Well-/dedifferentiated liposarcoma Synovial sarcoma	Underexpression	PRC1, PLK1, BCL2
*miR-145*	Liposarcoma	Underexpression	
*miR-152*	Leiomyosarcoma	Underexpression	MET, KIT
*miR-155*	Myxoid/round cell liposarcoma Dedifferentiated liposarcoma Pleomorphic liposarcoma	Overexpression	CK1α
*miR-17*	Synovial sarcoma	Overexpression	CDKN1A
*miR-183*	Synovial sarcoma	Overexpression	EGR1, PTEN
	Rhabdomyosarcoma	Overexpression	EGR1, PTEN
*miR-199b-5p*	Leiomyosarcoma	Underexpression	
	UPS	Overexpression	
*miR-206*	Rhabdomyosarcoma	Underexpression	CCND2, PAX3
*miR-21*	MPNST	Overexpression	PDCD4
*miR-26a-2*	Liposarcoma	Overexpression	RCBTB1, HOXA5
*miR-27a*	Rhabdomyosarcoma	Overexpression	RARA, RXRA
*miR-29*	Rhabdomyosarcoma	Underexpression	CCND2, PAX3, E2F7
	Fibrosarcoma	Underexpression	MMP2
*miR-29c*	MPNST	Underexpression	MMP2
*miR-30d*	MPNST	Underexpression	KPNB1
*miR-320a*	Leiomyosarcoma	Overexpression	
	UPS	Underexpression	
*miR-34a*	MPNST	Underexpression	
*miR-373*	Fibrosarcoma	Overexpression	SIRT1, mTOR
*miR-378a-3p*	Rhabdomyosarcoma	Underexpression	IGF1R
*miR-451*	Liposarcoma	Underexpression	
*miR-486*	Myxoid/round cell liposarcoma	Underexpression	PAI-1
*miR-497-5p*	Angiosarcoma	Underexpression	KCa3.1
*miR-520c*	Fibrosarcoma	Overexpression	SIRT1, mTOR

## Abbreviations

3′UTR	3′-untranslated region
BCL2	B-cell lymphoma 2
CCND2	Cyclin D2
CDK4	Cyclin dependent kinase 4
CDKN1a	Cyclin-dependent kinase 1
CK1α	Casein kinase 1α
CTX	Chemotherapy
DNMT1	DNA (cytosine-5)-methyltransferase-1
EGR1	Early growth response protein 1
EZH2	Enhancer of zeste homolog 2
HGF	Hepatocyte growth factor
HMGA2	High-mobility group AT-hook 2
HOXA5	Homeobox protein A5
IGF1R	Insulin-like growth factor receptor 1
KCa3.1	Potassium intermediate conductance calcium-activated channel
KPNB1	Importin subunit beta-1
MDM2	Mouse double minute 2 homolog
miRNA	Micro RNA
MMP-2	Matrix metalloproteinase-2
MPNST	Malignant peripheral nerve sheath tumour
mRNA	Messenger RNA
mTOR	Mechanistic target of rapamycin
NF-κb	Kappa-light-chain-enhancer of activated B cells
PAI-1	Plasminogen activator inhibitor-1
PDCD4	Programmed cell death protein 4
PLK1	Polo-like-kinase 1
PRC1	Protein regulator of cytokinesis
PRC2	Polycomb-repressor complex
Pre-miRNA	Precursor miRNA
Pri-miRNA	Primary miRNA
PTEN	Phosphatase and tensin homolog
RARE	Retinoic acid response element
RCBTB1	Regulator of chromosome condensation and BTB domain-containing protein 1
RTX	Radiotherapy
RXR	Retinoic X receptor
RXRA	Retinoic X receptor alpha
SIRT1	Surtuin 1
SMA	Smooth muscle actin
STS	Soft tissue sarcoma
THBS1	Throbmospondin 1
